# Ventricular Tachycardia Ablation Guided by Functional Substrate Mapping: Practices and Outcomes

**DOI:** 10.3390/jcdd9090288

**Published:** 2022-08-30

**Authors:** Sara Vázquez-Calvo, Ivo Roca-Luque, Andreu Porta-Sánchez

**Affiliations:** Arrhythmia Unit, Cardiology Deparment of the Institut Clínic Cardiovascular (ICCV) at Hospital Clínic de Barcelona, Universitat de Barcelona and Institut d’Investigacions Biomédiques August Pi i Sunyer (IDIBAPS), C/Villarroel No 170, 08036 Barcelona, Spain

**Keywords:** ventricular tachycardia ablation, functional mapping, hidden substrate, decremental conduction, deceleration zones

## Abstract

Catheter ablation of ventricular tachycardia has demonstrated its important role in the treatment of ventricular tachycardia in patients with structural cardiomyopathy. Conventional mapping techniques used to define the critical isthmus, such as activation mapping and entrainment, are limited by the non-inducibility of the clinical tachycardia or its poor hemodynamic tolerance. To overcome these limitations, a voltage mapping strategy based on bipolar electrograms peak to peak analysis was developed, but a low specificity (30%) for VT isthmus has been described with this approach. Functional mapping strategy relies on the analysis of the characteristics of the electrograms but also their propagation patterns and their response to extra-stimulus or alternative pacing wavefronts to define the targets for ablation. With this review, we aim to summarize the different functional mapping strategies described to date to identify ventricular arrhythmic substrate in patients with structural heart disease.

## 1. Introduction

Patients with structural heart disease that experience ventricular tachycardia (VT) have poorer outcomes compared to those who do not have VT [1,2]. Aiming to improve their prognosis has been a challenge over the last 30 years. Minimally invasive techniques for treating VT such as catheter ablation (CA) have an established role for such patients. Recent studies have shown as well favourable outcomes in terms of better prognosis and VT burden reduction [3,4,5]. Defining the critical regions sustaining VT had classically been performed by activation mapping and is considered the gold standard to identify the critical VT isthmus, but it is only tolerated in approximately 30% of patients. To overcome this problem, VT substrate mapping aiming to identify areas during stable rhythm (sinus rhythm or paced rhythm) that could be—but are not proven to be—involved in the VT circuit has gained widespread acceptance and some studies have shown superiority in terms of outcomes as compared to activation-mapping based VT ablation [6,7]. A simplistic way of depicting the VT substrate is by analyzing the peak-to-peak bipolar voltage amplitude, which has been correlated with the presence of myocardial scarring. By defining low-voltage areas (<0.5 mV), a border zone (0.5–1.5 mV), and healthy areas (>1.5 mV), several strategies were used to define the area to target with radiofrequency. Nevertheless, these thresholds have been obtained by conventional bipolar mapping catheters lacking contact sensors in the tip and validated in a small cohort of patients [8,9], so its application nowadays is controversial [10]. An alternative strategy is not just to quantify the peak-to-peak bipolar signal but to look for the qualities and timing of those signals, which have been described as late potentials [11] and their elimination shown to be useful in terms of decreasing episodes of VT during follow up [12,13,14]. Those RF targets are subject to some extent to interpretation and their assessment is limited by several factors: first, it depends on the capability of the mapping catheter to correctly detect the EGMs according to the electrode size, interelectrode spacing, and angle of the incoming wavefront to the bipolar pairs [15,16]. Concerning this matter, some studies have compared traditional point-by-point catheters with new high-density (HD) catheters showing a better discrimination of abnormal electrograms and better clinical outcomes with the HD catheters [17,18,19]. Second, it is known that the abnormal EGMs can be hidden within the far-field signal and occur during the surface QRS and not after it. Several strategies based mainly in different settings of pacing have been designed to unmask the substrate. Third, even if we could identify all the abnormal EGMs, these could lead to ablate large areas of myocardium not always related with VT circuits. Additionally, this could potentially create new areas with a slow conduction capability and generate new potentially conducting channels.

The objective of this review is to analyze the different strategies to identify and unmask the arrhythmogenic substrate apart from the findings of peak-to-peak voltage mapping, in order to improve VT ablation outcomes.

## 2. Our Definition of Functional Mapping

We propose the definition of VT functional substrate mapping as a grouping term that uses not only the peak-to-peak voltage of the EGMs to analyze them but also their propagation patterns within the low-voltage areas and its response to either varying wavefronts of the activation or varying cycle lengths of pacing or extra-stimulation. Table 1 summarizes the different techniques.

## 3. Techniques for VT Functional Substrate Mapping

### 3.1. Decrement Evoked Potential (DEEP) Mapping

Voltage channels can be easily identified in patients with VT through voltage maps; adjusting the thresholds but a low specificity (30%) for VT isthmus has been described with this approach. The presence of LPs inside the CCs increases the specificity for identifying critical VT termination sites (85%) [20]. Despite strategies focusing on complete LP abolition, inducibility at the end of the procedure continues to be high (40%) and the VT recurrence still remains around 30% after one-year follow up [21,22].

**Figure 1:**
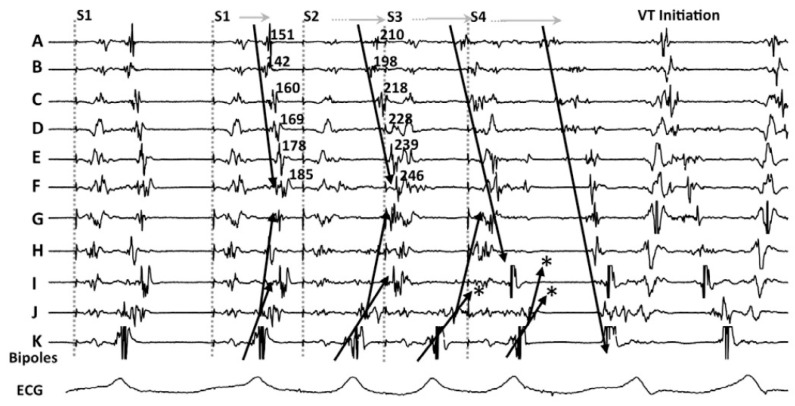
An example of decremental conduction, unidirectional block, and induction of ventricular tachycardia (VT) from Jackson et al. [25]. During right ventricular pacing, local abnormal potentials can be seen on bipoles A to K. With the introduction of the first extrastimulus (S2), an important delay in bipoles A to J is observed. With S3, there is a block (*) of the local potential on bipole I and subsequently with S4 there is block (*) at the VT exit site (bipole J). Block at the VT exit site and conduction delay through the entrance of the channel set the basis for re-entrance. The mechanistic study by Jackson et al. showed that those late potentials that had decremental properties were more frequently co-localized with the VT isthmus.

Looking in depth into the mechanism of re-entry, which is the main mechanism of VT in patients with heart disease, conduction delay preceding unidirectional block has been demonstrated as essential for its initiation and maintenance [23,24] (Figure 1).

In their seminal paper, Jackson et al. [25] hypothesized that ventricular EGMs which showed decremental conduction (called decrement evoked potentials or DEEPs) could be more likely to colocalize with critical VT circuits than LPs without decremental conduction. They retrospectively analyzed the results of intraoperative mapping from six ischemic patients, and after that, used a human ventricular myocardium ionic model [26] to mathematically correlate DEEPs with the diastolic isthmus of VT. The mapping protocol consisted in performing a RV pacing train at 600 ms with a single extra-stimulus delivered at the ventricular effective refractory period (VERP)+20 ms whenever an LP or fractionated potential was identified. If the local potential delayed >10 ms, this would be annotated as a DEEP. In this setting, the use of DEEP mapping identified the diastolic pathway with greater specificity than LP mapping with similar sensitivity. Of note, only areas showing LP during baseline conditions were analyzed for the presence of DEEPs. 

These results were confirmed in the clinical setting by Porta-Sanchez et al. [27] with 20 consecutive patients with ischemic cardiomyopathy in a multicentric study. They identified DEEPs as those LPs with decremental activity (>10 ms delay after pacing protocol). The mean area with LPS was 16.8% of the myocardium mapped vs. 4.8% with DEEP (*p* < 0.001), but areas with DEEPs performed better than LPs at colocalizing within VT isthmus (ROC area under the curve of 0.86 vs. 0.79 with LPs). The ablation strategy consisted in ablation only of the DEEP regions and only expanded the ablation if the patient was still inducible (20%). Recurrence rates after DEEP-guided ablation were similar to what has been described in the literature (75% VT freedom at 6 months).

### 3.2. Hidden Slow Conduction (HSC) Mapping

**Figure 2:**
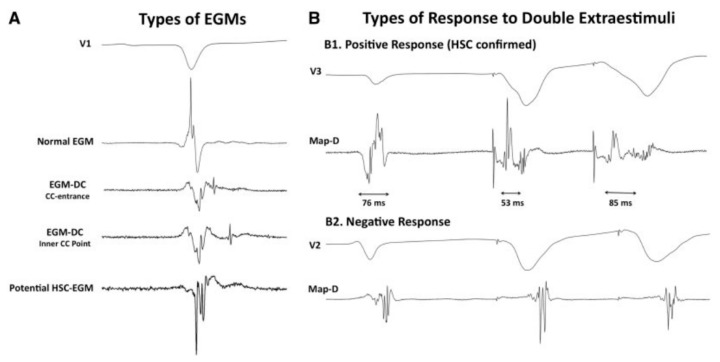
Electrogram classification and response to a double extraestimulus from Acosta et al. [28]. (**A**) different types of EGMs are shown: from normal through CC-EGMs to potential HSC. (**B**) the response of the potential HSC-EGM after a double extraestimulus is represented, considering a positive response if the local potential is delayed. Ablation was undertaken in those regions feeding the preserved voltage channels with the “scar dechanneling” technique.

Acosta et al. [28] described a new method to unmask EGMs showing hidden slow conduction (HSC-EGM) using a double ventricular extra-stimulus in 37 consecutive patients (with ischemic and non-ischemic myocardiopathy). They distinguished four different EGMs: normal (less than three sharp deflections with amplitude >3 mV and duration <70 ms; fractionated (multiple deflections, amplitude <0.5 mV and duration >133 ms; late (any EGM lasting beyond the QRS); and potential HSC-EGM (>3 deflections but <133 ms). Whenever a potential HSC-EGM was identified, a double extra-stimulus from the RV apex was delivered at VERP+60 ms and VERP+40 to 20 ms, respectively. When the EGM split and delayed from the far-field signal, it was annotated as HSC-EGM (Figure 2). These EGMs were found in 56.7% of patients. Ablation was delivered at conducting channels (scar dechannelling technique) and HSC-EGMs. Interestingly, patients showing HSC-EGM were more frequently ischemic with smaller and more heterogeneous scar areas and had less VT inducibility after ablation compared with an historic cohort. This study was enlarged by the same authors with a publication in 2020 including 70 patients and reproducing similar conclusions and potentially pointing towards a better outcome in terms of VT freedom [29].

### 3.3. Evoked Delayed Potential (EDP) Mapping

De Riva et al. [30] performed a similar study to Acosta’s and Porta-Sánchez’s including 60 consecutive ischemic patients and found that 62% of patients had hidden slow conduction (unmasked by pacing RV 500 ms + a single extra-stimulus VERP+50 ms and defined as conduction delay > 10 ms or block). These patients had better left ventricular function and smaller scar areas on the electroanatomical mapping system. The authors hypothesized that an ablation strategy based on the exclusive elimination of the EDPs can lead to a lower incidence of VT recurrence in the follow up (compared with an LVEF-matched historical cohort from their own institution). 

In both Acosta’s and De Riva’s studies, the distribution of hidden substrate was analyzed and, when possible, compared not only with voltage maps but also cardiac magnetic resonance (CMR). Nearly 20–35% of these EGMs were located in healthy tissue (>1.5 mV bipolar peak to peak) as measured by mapping. This number clearly decreased when the analysis was made by CMR (8–9%), which emphasizes the possibility of a better definition of the VT substrate by CMR and functional substrate mapping as compared to the conventional voltage map. This is consistent with the study by Oduneye [31], in which a real-time CMR-guided electrophysiology system was used observing that abnormal EGMs were seen more often in BZ defined by CMR. One possible explanation for this inaccuracy of the voltage map is that far-field healthy-tissue signals can obscure the near field signals underestimating the scar and hiding the abnormal EGMs from conducting channels, especially in patients with small areas of scar. In those patients, trying to unmask the hidden substrate could be especially relevant but needs to be performed in a systematic way throughout the entire substrate. 

### 3.4. Paced Electrogram Feature Analysis (PEFA)

A better characterization of the EGM response after a close-coupled extra-stimuli was studied by Redfearn et al. [32] both in ischemic and control subjects, defining four different types of response (type 0: no change in the characteristics of the EGM; type I: increased in duration and latency; type II: increase in duration; and type III: increase in latency) and correlated them with the VT isthmus. Programmed stimulation was performed using a RV stimulation 600 ms followed by an extra-stimulus applied every 7th beat: VERP+150 ms, VERP+100 ms, VERP+50 ms. So, three different maps were generated. They observed that the mean duration of the EGMs increased in all patients (significantly more in heart disease patients), but the latency behaved differently: it decreased with the first extra-stimuli (VERP+150 ms) and then (with VERP+100 and VERP+50 ms) increased in healthy patients, but continuously increased in all ischemic population. They also identified that latency (type III) was a common response in both LAVA and non-LAVA areas, type I and II responses found most frequently at VT termination sites. 

The PEFA strategy was tested with 10 ischemic patients, targeting type I and II EGMs for radiofrequency, obtaining longer procedures but a lower VT inducibility rate at the end of the procedure in the interventional group as compared to the derivation cohort.

### 3.5. The Barts Sense Protocol

In an elegant study, focusing on a mechanistic and physiopathologically sound hypothesis, Srinivasan et al. designed an innovative strategy. It is based on the findings by Roelke et al. [33] that found that the VT was most often preceded by late-coupled premature depolarizations. Some years after that, Saeed confirmed these results showing that 66% of VTs were initiated after a single extrasystolic beat [34].

**Figure 3:**
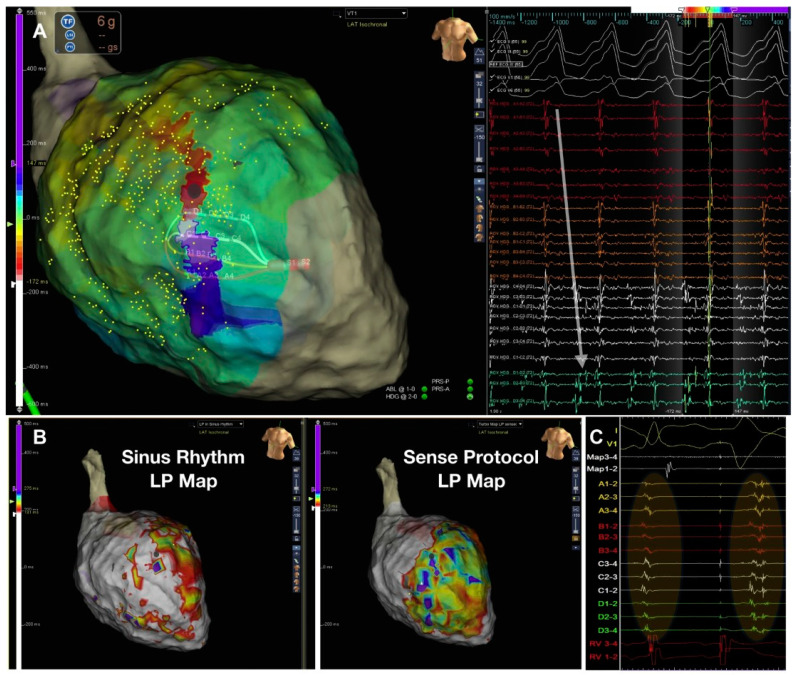
Figure adapted from Srinivasan et al. [35] illustrating the Barts’ sensed protocol. (**A**) shows a high-density VT activation map. (**B**) illustrates the 2 activation maps during sinus rhythm (left) with a late potential (LP) color timing map and the activation time during the sensed RV pacing beat ((**B**) right) showing a greater region of LPs during Bart-sense-protocol colocalizing to a greater extent with the mapped isthmus of the induced VT (**A**). (**C**) shows delay and splitting of LPs during sense protocol (second beat) is observed within the region of the diastolic pathway of VT.

With the idea of closely reproducing this mechanism, the Barts Sense Protocol was designed [35]. A cohort of 30 ischemic patients were prospectively included for VT ablation in two UK centres. Two substrate maps were obtained, one during sinus rhythm and another after pacing the RV every fifth beat to simulate an extra-stimulus close to VERP. There were no significant differences in the scar area when comparing the intrinsic sinus rhythm map vs. the sensed extrasystole maps, but larger areas of late activation were identified after the extrasystole (Figure 3). In 21 patients, 75 VTs were analyzed with high-density activation mapping. This allowed to illustrate that, in 80% of cases, these abnormal EGMs were situated within 10 mm of the critical VT isthmus. Those stable and mappable VTs are not always the most common presentation of VTs in such advanced substrates, but it allowed to highlight that the main advantage of that strategy compared with DEEP mapping and hidden substrate mapping is that is could be slightly more automated, with less manual reannotation of EGMs with a reasonable balance of mean RF time (approximately 30 min).

### 3.6. PHYSIO-VT

**Figure 4:**
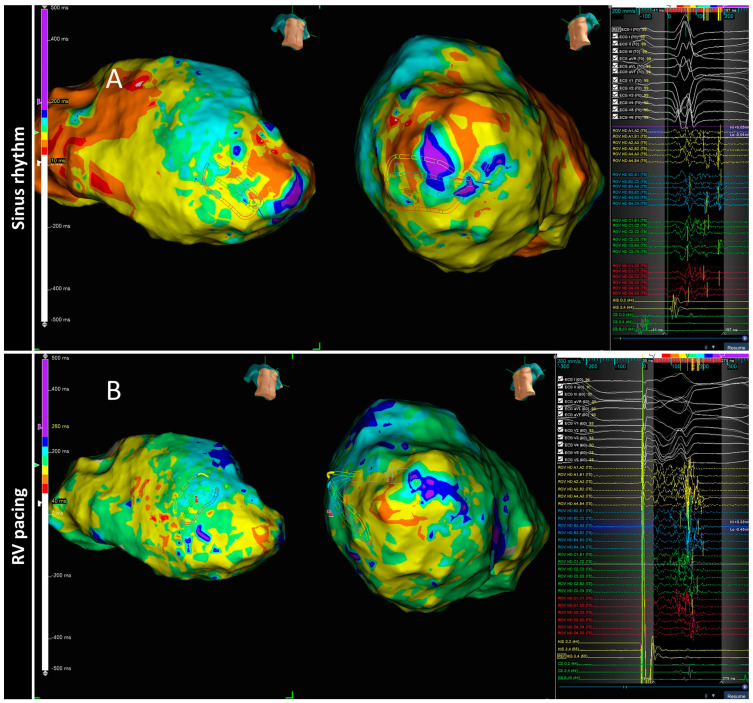
The spatial distribution of activation slowing is influenced by the direction of left ventricular activation. An example of two different maps performed in the same patient during two different ventricular activations: spontaneous sinus rhythm (**A**) and RV pacing (**B**). The general zone of activation slowing was similar between maps, similar to the signals shown on the right side of the panels obtained at each HD grid catheter location depicted, consequently, this area should be ablated according to the PHISIO-VT study [38].

Conducting channels responsible for VT circuits are based mainly on anatomical features consisting of replacement of cardiomyocytes with fibrosis and persistence of viable cells inside the scar capable of slow conduction. However, the sole presence of scar is insufficient to produce the electroanatomical setting for re-entry. Several other factors, such as cellular coupling, gap junction distribution and function and fiber disarray, can lead to a nonuniform anisotropic conduction [36,37]; whether this is manifested and present independently of the rhythm that the patient presents was the main question for the PHYSIO-VT study. This study, led by Anter E et al., focused on analyzing the location of deceleration zones as assessed by the varying possible wavefronts and whether they identified overlapping areas to target with RF [38]. It was a multicenter study with 85 ischemic patients who underwent VT ablation. Ultra-high density left ventricular mapping was performed during activation from 3 different wavefronts: SR, RV and LV pacing at 600 ms. Activation mapping during SR was performed in 90.5% patients, during RV or LV in 95.3% and from all 3 directions in 43.5%. In 43.7% patients with left bundle branch block, LV activation during SR was very similar to activation from RV, so in patient with left bundle branch block, an activation map was performed only from SR and LV. The main result was that activation from RV or LV allowed to unmask additional areas of activation slowing as compared with SR maps (mapping during SR identified only 66.2% of all activation slowing) (Figure 4). The total “accumulative slowing area” detected using the 3 different wavefronts was targeted for ablation and interestingly those patients in whom RV and LV mapping was performed in addition to SR mapping presented less incidence of appropriate ICD therapies in the follow up.

### 3.7. Isochronal Late Activation Mapping (ILAM)

In 2015, a new substrate-based ablation strategy called ILAM (isochronal late activation mapping) was developed by Irie et al. [39]. These local activation time maps are performed during sinus rhythm (or RV pacing) and based their local activation time annotation on the latest part of the EGM, creating a map with a window starting at the earliest region of activation and ending at the latest site of activation. The overall activation time is divided into eight isochrones represented with 8 different colors, so the thickness of an isochrone is a graphical representation of the conduction velocity (distance/time). In the mechanistic study, Irie et al. [39] found that with that approach, the most likely regions to correlate with the VT isthmus were not the area with the latest activation but slow conduction regions propagating into the latest zone of activation using such an annotation criterion.

**Figure 5:**
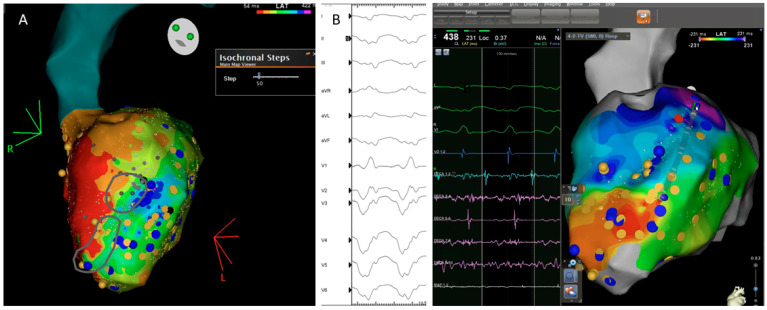
Correlation between the ventricular tachycardia (VT) circuit with critical diastolic pathway (**A**) and deceleration zone (DZ) location during sinus rhythm as depicted during ILAM mapping (**B**). Late isochronal activation map is shown in the left side of the panel (8 isochrones). Two deceleration zones are observed (more than 3 isochrones in less than 1 cm radius) in the anterior wall. The termination site of VT (on the right) colocalized to the DZs.

Subsequently, in 2019, Aziz et al. [40] presented a study with 120 patients who underwent VT ablation following ILAM approach and identified as relevant the deceleration zones (DZ) that were defined as regions with three different colors in less than 1 cm radius. In most instances (76%), mapping was performed during sinus rhythm and an average of 2 ± 1 DZ were identified. In cases of several DZs, the protocol encouraged to only ablate the primary DZ and check if the patient remained noninducible. In this study, DZs demonstrated a high correlation with VT isthmus, colocalized to successful VT termination sites in 95% of cases (Figure 5) and VT freedom at 12 ± 10 months of follow up was 70%, with ischemic patients having better VT-freedom (80%) than non-ischemic patients (63%).

## 4. Discussion

VT ablation remains a challenging procedure due to the high recurrence rate and the complex subset of patients experiencing such a life-threatening condition. Voltage maps have initially constituted an alternative to the difficulty of mapping VT due to hemodynamic instability allowing, with the classic thresholds of 0.5–1.5 mV, to characterize the scar and “visualize” the surviving myocardium without the need to induce VT. The low specificity of these voltage channels to accurately identify the VT isthmuses [20] has led to an extensive study of EGMs, especially within the scar, identifying several patterns of so-called late potentials [41] and LAVAs. Elimination of these EGMs has been associated with less VT recurrences in the follow up [12]. Different substrate ablation approaches have been suggested since then, from extensive ablation (scar homogenization) [42] to a more limited method as scar dechanneling [7], but recurrence rate continued to be non-neglegible [21]. In this sense, VT functional substrate mapping strategies based in the concept of a non-static behavior of the arrhythmic substrate have evolved with the aim to improve outcomes and identify targets for RF that could have been missed with previous “static” mapping. The main findings of the functional VT substrate mappping approaches can be summarized as follows:

1. The arrhythmic substrate is dynamic: its electric properties can change with different pacing settings and that help unmask regions that are critical for re-entry.
  - Late potentials showing decremental properties (DEEPs) seem to be more likely associated with VT isthmuses.
  - A pace protocol based in ventricular extra-stimulus has demonstrated to be able to change the shape and duration of selected EGMs, especially in patients with small scars. Targeting those electrograms that delay from the far field signal could lead to less VT recurrence in the follow up.
  - The direction of the wavefront also has an important impact in the arrhythmic substrate characterization and needs careful evaluation in some patients.
2. The analysis of the timing of the electrograms, not absolute and individually, but relative to the duration of all the ventricular electrograms and their pattern of propagation, has led to the development of a method for the identification of deceleration zones. These DZs have shown a good correlation with VT isthmus.

### 4.1. Additional Value as Compared to Historical VT Cohorts

VT functional substrate mapping strategies have let us increase the knowledge about the arrhythmic substrate, which in itself represents an important advance. Understanding the arrhythmic substrate as something not static but functional has been shown to be important to improve ablation results. Among the most outstanding findings, using different paced protocols and extra-stimulation, is the definition of new electrograms called DEEPs. These DEEPs have showed to have a greater specificity for identifying VT isthmus compared with conventional non-decremental LPs. The basis for pursuing those potentials with decremental conduction lies in how the ventricular tachycardias in scar-related patients are initiated [23,24]. Multiple studies have shown that this mechanism occurs after a conduction delay (or decrement) preceding unidirectional block and leading to reentry. It is reasonable, therefore, to think that those potentials with decremental capacity are responsible for the arrhythmic circuits.

Similarly, the main mechanism responsible for generating this decremental conduction and inducing VT is the extrastimuli [33,34], so performing map pacing with a stable rhythm might not be the most thorough way to identify all the potentially arrhythmogenic properties of the tissue. The different studies that have tried to define the behavior of the substrate after ventricular extra-stimuli have led to separation of the near-field signal from the far-field signal and the identification of hidden substrate that can potentially be ablated. 

With regards to the direction of LV activation, several studies agreed on the greater accuracy to identify LP and LAVAs with an RV and/or LV pacing protocol compared with sinus rhythm. More specifically, it seems that the maximal activation slowing is achieved pacing close to the site where the wavefront first interacts with the infarcted area [38].

In this sense, one of the main messages behind functional VT mapping strategies is that by varying the speed and origin of the stimulation, we change the properties and characteristics of the substrate. Therefore, if we exclusively perform an electroanatomic map in sinus rhythm, without using extra-stimuli, we will have to assume a partial and not complete information about the substrate. 

A second important message comes from the perspective of understanding the behavior of electrograms not only by themselves but relating them to neighboring electrograms. DZs are defined as areas not only with delay (that can importantly vary depending on pacing location, i.e., if we pace from the right ventricular apex, the most delayed activation will occur naturally in the lateral wall of the left ventricle) but deceleration. The ILAM has emerged as a widespread method due to automatic annotation, visual representation of areas of interest and, of course, the higher correlation between DZs and VT isthmus [39], allowing more limited ablation targets.

Despite the important advances shown with functional VT substrate mapping, some limitations must be addressed. First, none of the previous studies focused specifically on the behavior of intramural substrates during functional mapping strategies. This could also be due to the lack of pre-procedural CMR to define the intramural substrate. Undoubtedly, identifying the septal arrhythmic substrate during mapping from both RV and LV endocardium is of importance, and functional mapping of the area and its comparison with a stable rhythm can help define targets for RF [43]. The role of systematic functional mapping in this setting remains unknown. Additionally, non-ischemic substrate tends to be located preferentially in anteroseptal locations, which is an area particularly refractory to RF delivery and substrate elimination. Most of the functional VT substrate studies have included ischemic patients, so the results cannot be readily extrapolated to the non-ischemic population, with a more patchy and non-confluent fibrosis that has always shown poorer outcomes after ablation [1].

### 4.2. Functional Substrate and High Density Mapping: Ablate More or Ablate Less?

The substrate approach strategy is based on the correct identification of the arrhythmic substrate. The bipolar signal depends on the electrode size, interelectrode spacing, and angle of the incoming wavefront to the mapping catheter, so areas of low-amplitude EGMs as LPs or LAVAs cannot be detected with classical bipolar catheters. High-density catheters have been shown to facilitate the discrimination of LAVAs/LPs, providing a more detailed characterization of the arrhythmic substrate, which is even more important in low-voltage areas [18,44]. 

Interestingly, less VT recurrence has been reported using this type of catheter [14,17], although the only randomized comparison failed to show an important difference in outcome [45].

Likewise, the functional substrate mapping can help us to unmask the hidden substrate both with extra-stimulus techniques and pacing from different locations, increasing the amount of potentially arrhythmic substrate. Furthermore, this strategy allows to better identify areas more specifically related with VT isthmus, such as DEEPs [27] or DZs [39]. 

The fact that we can visualize a greater amount of arrhythmic substrate but that at the same time we have tools to better delimit which zones are associated with critical VT circuits has led to some controversy nowadays regarding the preferred ablation strategy.

One possibility is based on performing limited ablation focused on identifying surrogates of the VT isthmus, either using activation mapping if possible, and/or targeting DZs or DEEPs (highly associated with critical isthmus). This could decrease the procedure and radiofrequency times at the expense of leaving an undetermined amount of arrhythmic substrate unablated, which could be responsible for events in the future. 

The other perspective relies on a larger ablation approach involving all of the arrhythmic substrate. Techniques such as scar homogenization based on the elimination of the entire substrate have demonstrated less VT inducibility at the end of the procedure and possibly a decrease in VT recurrence rate during follow up [42].

### 4.3. Current Practice and Future Directions

Advancements in EAM systems and multielectrode catheters have allowed for a semi-automatization of the mapping process with different rhythms and thus have been instrumental in the description and analysis of such functional techniques. All of the most common mapping systems can be configured to allow for the creation in real time of several maps while the catheter is roving the chamber of interest or by replaying the movements and signals of the catheter afterwards and recreating a new map. Those features are present both in the CARTO v3 system (TM), Ensite X (TM) and Rhythmia HDX mapping systems (TM).

A unified and standardized VT ablation strategy has not yet been defined with total reproducibility such as in other standard EP procedures. The lack of randomized studies, the low number of patients included in them, the evolution of the substrate, the difficulty in creating transmural lesions, and several other factors make it difficult to draw clear conclusions about the different mapping methods, so there currently exists a great variability between centers and operators who perform VT ablation procedures.

A multicenter randomized study focused on performing the best possible analysis of the arrhythmic substrate (including the use of high-density mapping catheters, several pacing locations and an extra-stimulus protocol to identify hidden substrate with and without decremental properties and probably the image integration) and then test a more limited or more extensive ablation strategy could lead to clarification of the best approach for VT ablation and eventually improve clinical outcomes.

## Figures and Tables

**Table 1:** Comparation of different VT functional mapping strategies.

Strategy	Article	Population	Mapping System	Stimulation Setting	Measurement	Objective	RF Target	RF Time (min)	Results
**DEEP**	JACKSON 2015	6 ischemic.	Intraoperative mapping: custom-made 112 electrode ballon	If LP of fractionated EGM are identify: RV pacing 600 ms + VERP+20 ms	DEEP: delayed local potential after stimulation	To compare DEEP vs. LP mapping to identify VT isthmus (retrospectively)	VT critical sites based on activation mapping	N/A (surgical cryoablation)	DEEP mapping was more specific than LP mapping for identifying VT isthmus.
	PORTA-SÁNCHEZ 2018	20 ischemic.	CARTO: 9 Decanav 6 Pentarray 4 ablation cath.	For all LPs: RV pacing 600 ms + VERP+20 ms	DEEP: S2 local potential delayes or splits > 10 ms compared with S1	To compare DEEP vs. LP mapping to identify VT isthmus	DEEP area	30.6	Specificy of DEEP to detect VT isthmus was better than LPs
**HIDDEN SUBSTRATE**	ACOSTA 2015	37 patients: 75.7% ischemic.	CARTO	Identify potential HSC-EGM (>3 deflections and <133 ms) and double extra VERP+60 and VERP+40 to 20 ms	HSC-EGM: potential HSC-EGM that delays after stimulation	To analyses characteristics of patients with HSC-EGM	CCs (scar dechanneling) and HSC-EGM	Interv. group: 17.41 Hist. cohort: 23.11	Patients with HSC-EGM: More frequently ischaemic, smaller low voltage area, low number of LPs Location of HSC-EGM: EAM: 18.2% scar area vs. CMR: 92% scar area
	DE RIVA 2018	60 ischemic.	CARTO	RV pacing 500 MS + single extra VERP+50 ms	EDP: low amplitude (<1.5) near field potentials with conduction delay > 10 ms or block.	To compare patients with hidden vs. not hidden substrate	EDPs	Interv. group: 15 Matched cohort: 13	Hidden substrate group: Better FEVI, smaller scar and dense scar, higher 12 m VT free survival
**PEFA**	REDFEARN 2018	(1) 14 ischemic. and 5 healthy controls (2) 10 ischemic	Ensite Precision	RV pacing 600 ms(x6) + VERP 150 RV pacing 600 ms(x6) + VERP 100 RV pacing 600 ms(x6) + VERP 50	4 types of response related to latency and EGM duration	(1) To compare different EGM responses after stimulation protocol (2) To validate PEFA method	(1) Operators were blinded to PEFA (2) Type I and II	Interv group: 39.47 Cohort: 39.88	(1) Type I and II responses: most frequently at VT termination sites (2) PEFA approach reduced VT inducibility
**BARTS**	SRINIVA-SAN 2018	30 ischemic.	Ensite Precision	Sinus rhythm (SR)(x5) + VERP 20 ms (SP)	Annotation of LP and LAVAs	To compare LP/LAVA with VT isthmus in two different maps: SR and SP	Total LPs and LAVAs	32	LP/LAVAs observed during SP were able to identify regions critical for VT ablation with a greater accuracy than SR mapping
**PHYSIO VT**	ANTER 2020	85 ischemic.	RHYTMIA 92.8% CARTO 7.2%	-SR and RV Pacing 600 ms and LV Pacing 600 ms	Area of activation maps (isochronal maps of 10 ms steps)	To compare areas of activation slowing and critical VT isthmus in three different maps (SR, RV and LV)	Acumulative area of activation slowing	27.7	The direction of LV activation is influenced by the magnitude and location of activation slowing: SR Mapping identify 66.2% of the entire area of activation slowing. RV and LV unmask an additional 33%
**ILAMS**	AZIZ 2019	120 patients: 50% ischemic	Ensite Precision	Annotation of last deflection and division of the total activation window in 8 equal isochrones	Deceleration zones (DZ): 3 isochrones in less than 1 cm.	To correlate DZ with VT isthmus	Primary DZs	29	DZs identify during SR are strongly predictive of critical sites for reentry.

## Data Availability

Not applicable.
